# Light and Circadian Signaling Pathway in Pregnancy: Programming of Adult Health and Disease

**DOI:** 10.3390/ijms21062232

**Published:** 2020-03-23

**Authors:** Chien-Ning Hsu, You-Lin Tain

**Affiliations:** 1 Department of Pharmacy, Kaohsiung Chang Gung Memorial Hospital, Kaohsiung 833, Taiwan; chien_ning_hsu@hotmail.com; 2 School of Pharmacy, Kaohsiung Medical University, Kaohsiung 807, Taiwan; 3 Department of Pediatrics, Kaohsiung Chang Gung Memorial Hospital and Chang Gung University College of Medicine, Kaohsiung 833, Taiwan

**Keywords:** circadian rhythm, developmental origins of health and disease (DOHaD), developmental programming, glucocorticoid, hypertension, light, melatonin, pregnancy

## Abstract

Light is a crucial environmental signal that affects elements of human health, including the entrainment of circadian rhythms. A suboptimal environment during pregnancy can increase the risk of offspring developing a wide range of chronic diseases in later life. Circadian rhythm disruption in pregnant women may have deleterious consequences for their progeny. In the modern world, maternal chronodisruption can be caused by shift work, jet travel across time zones, mistimed eating, and excessive artificial light exposure at night. However, the impact of maternal chronodisruption on the developmental programming of various chronic diseases remains largely unknown. In this review, we outline the impact of light, the circadian clock, and circadian signaling pathways in pregnancy and fetal development. Additionally, we show how to induce maternal chronodisruption in animal models, examine emerging research demonstrating long-term negative implications for offspring health following maternal chronodisruption, and summarize current evidence related to light and circadian signaling pathway targeted therapies in pregnancy to prevent the development of chronic diseases in offspring.

## 1. Introduction

Light provides much of the information that enables organisms to adapt to their environment. When sunlight is unavailable at night and/or indoors, artificial light enables humans to see and permits productivity [1]. However, exposure to excessive and obtrusive light produced by humans at night results in light pollution [1]. Global light pollution has been increasing exponentially during the past century [1]. Light pollution can cause unintended physiological consequences. In mammals, the production of melatonin—a key player in circadian regulation—can be suppressed by light [2]. Light also activates the adrenal glands and induces a surge in glucocorticoid (GC) levels via the suprachiasmatic nucleus (SCN), part of the sympathetic nervous system [3]. Light pollution disrupts circadian rhythms by altering downstream signaling pathways and can lead to the development of a variety of chronic diseases, such as cancer, cardiovascular disease, diabetes, metabolic syndrome, and obesity [4,5,6,7]. Conversely, the use of light therapy has been evaluated in jet lag, shift work, and circadian rhythm disorders [8,9]. Further, melatonin treatment confers a number of beneficial effects to human health, including those related to cardiovascular function, blood pressure, the nervous system, and lipid and glucose metabolism [10,11,12].

Most chronic diseases originate from early life. The developing fetus, if exposed to a suboptimal environment during pregnancy, experiences alterations to normal patterns of growth and development that increase its vulnerability to a wide range of chronic diseases in later life [13]. This concept is called the developmental origins of health and disease, or DOHaD [14]. The developing fetal circadian system can be programmed by the external environment. However, the impact of light-related chronodisruption during pregnancy on offspring health remains poorly understood. 

This review included publications on the relationship between the light and circadian signaling pathway and pregnancy and the effects on the developmental programming of chronic diseases in offspring. We searched the PubMed/MEDLINE databases for studies published in English between January 1990 and January 2020, using the following search terms: “light”, “circadian rhythm”, “chronodisruption”, “clock gene”, “melatonin”, “pineal”, “glucocorticoid”, “developmental programming”, “DOHaD”, “offspring”, “progeny”, “cardiovascular diseases”, “obesity”, “hypertension”, “neurological diseases”, “pregnancy”, “mother”, “maternal”, “gestation”, “neonatal”, “perinatal”, “light therapy”, “photobiomodulation”, and “reprogramming.”. Relevant studies were assessed for inclusion by title and abstract, followed by full-text review.

In this review, we particularly focused on the following areas: the impacts of light and circadian signals in pregnancy and fetal development; human studies for the programming of adult diseases related to maternal chronodisruption; animal models of maternal chronodisruption; mechanisms underlying maternal chronodisruption-induced programmed diseases; and targeting of the light and circadian signaling pathway as a reprogramming therapy to prevent DOHaD-related diseases.

## 2. The Impacts of Light and Circadian Signals in Pregnancy and Fetal Development

### 2.1. The Retinohypothalamic Pathway

**Figure 1:**
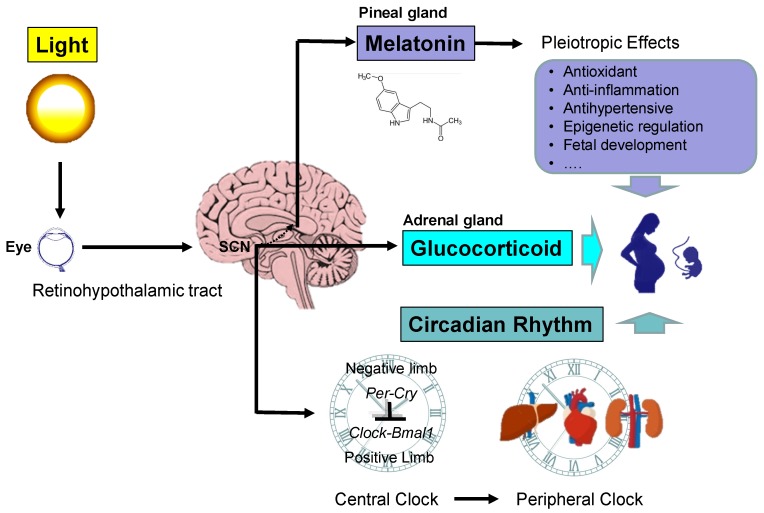
Schema outlining the light and circadian signaling pathway in pregnancy. The retinohypothalamic tract transmits light from the eyes to the suprachiasmatic nucleus (SCN). In the pineal gland, melatonin synthesis follows a rhythm driven by the SCN. Melatonin has pleiotropic biological function whereby it regulates pregnancy and fetal development. The SCN also stimulates the release of glucocorticoids (GCs) in the adrenal gland in a light-dependent manner. GC signaling is crucial for fetal development. The circadian clock system consists of central and peripheral clocks, which are coordinated to produce daily rhythms. The molecular mechanisms responsible for the generation of the rhythmicity within the SCN and peripheral clocks are regulated by interactive transcriptional–translational feedback loops between the clock genes, including *Per1, Per1, Cry1, Cry2, CLOCK, BMAL1, Rev-erbα*, and *CK1ε*, and their protein products. The cellular circadian clock consists of positive and negative interlinked regulatory limbs. CLOCK and BMAL1 form a heterodimer that positively activates the rhythmic expression of *Per, Cry*, and *Rev-erbα* genes. The negative limb includes the PER and CRY proteins. After nuclear translocation of both proteins, the PER:CRY complex interacts with the CLOCK:BMAL1 heterodimer and inhibits CLOCK:BMAL1-mediated transcription. The regulation of Bmal1 transcription is mediated mostly by REV-ERBα. The SCN and peripheral clocks operate with similar components and share a similar molecular core clock mechanism. Circadian signals can transfer from mother to fetus. The rhythms of melatonin and GCs provide synchronization signals for peripheral clocks. The interactions between light, the circadian clock, and the circadian signals melatonin and GCs in pregnancy ultimately impact the health of the mother and offspring.

Light often serves as a stimulus to induce circadian clock responses. Circadian systems detect light and transform it into a timed signal that synchronizes all physiological processes to the same zeitgeber. This system is driven by the circadian clock, which is composed of an intrinsic pacemaker, the light input by photoreceptors, and output signals [15]. Circadian clocks consist of a central clock within the SCN and peripheral clocks within peripheral organs. As shown in Figure 1, there is a connection called the retinohypothalamic tract (RHT) that extends from the retina of the eye to the SCN [16]. In the presence of light, neurons of the SCN directly inhibit the neurons in the paraventricular hypothalamic nucleus (PVN) that are responsible for stimulating the pineal gland to secrete melatonin. That is, light inhibits melatonin secretion, whereas darkness allows the pineal gland to secrete melatonin [17]. Melatonin is synthesized during the dark period of the daily light:dark cycle, independent of whether the animal is diurnally or nocturnally active. The melatonin secreted by the pineal gland provides inhibitory feedback information to SCN neurons. Peripheral clocks are present in nearly every tissue and organ system (i.e., liver, heart, and kidney), which are regulated by the SCN through the autonomic nervous system (ANS) and rhythmic entraining signals (Figure 1). 

There is a strong interaction between the circadian clock system and the hypothalamic–pituitary–adrenal (HPA) axis [18]. The light-activated central clock controls the HPA axis through synapses between the SCN and the PVN, thereby inducing the release of adrenal GCs. Circulating GCs act as a strong entrainment signal for peripheral circadian oscillators. GCs play a dual role as a circadian and stress signal. In the absence of stress, GC secretion displays a circadian rhythm, with rise prior to wake up. The coupling of GC secretion with activity phase onset allows GC to mediate similar physiological functions regardless of whether in diurnal species is primarily active during the day (e.g., humans) or the night in nocturnal species (e.g., rodents) [18]. On the other hand, a stressful event results in secretion of GCs. SCN uses both humoral and neuronal pathways to transmit time-related information to peripheral clocks. The rhythms of melatonin and GCs provide synchronization signals for clocks in peripheral organs. 

### 2.2. Maternal and Fetal Circadian Rhythms

The basic molecular core clock mechanism in cells responsible for the generation of rhythmicity within the SCN and peripheral clocks is formed by interactive transcriptional–translational feedback loops between the clock genes, namely two *Per* (encoding the period, Per1,2), two *Cry* (encoding the cryptochrome, Cry1,2), circadian l ocomotor output cycles kaput (CLOCK), brain and muscle aryl-hydrocarbon receptor nuclear translocator-like 1 (BMAL1), *Rev-erbα* and casein kinase 1 epsilon (CK1ε), and their protein products [19,20]. The cellular circadian clock consists of positive and negative interlinked regulatory limbs [19,20]. CLOCK and BMAL1 form a heterodimer and positively activate the rhythmic expression of *Per*, *Cry*, and *Rev-erbα* genes. The negative limb includes the PER and CRY proteins. After nuclear translocation of both proteins, the PER:CRY complex directly interacts with the CLOCK:BMAL1 heterodimer and inhibits CLOCK:BMAL1-mediated transcription. The regulation of *Bmal1* transcription is mediated mostly by REV-ERB. The SCN and peripheral clocks operate with similar components and share a similar molecular core clock mechanism. *Clock* genes are expressed in the oocytes and in fetal organs during organogenesis, while they are not involved in circadian clock regulation in oocytes [21].

### 2.3. Ontogenesis of the Circadian Clock

In mammals, the maternal circadian clock within the SCN is entrained by photic as well as non-photic cues with the time of the day [22]. The *Per1* mRNA is upregulated by photic and downregulated by non-photic entraining stimuli. The fetal SCN and peripheral clocks are entrained via, as yet only partially recognized, rhythmically delivered maternal stimuli. Ontogenesis of the mammalian fetal SCN clock is a gradual process that occurs from the fetal to postnatal periods [23]. First, SCN neurogenesis occurs from 31 weeks of gestation in primates [24]. Second, the SCN is innervated by the RHT and is responsive to light at birth in mammals [25]. Finally, a mature mammalian circadian system displaying overt physiological rhythms slowly develops throughout the late prenatal and early postnatal period. Although the mammalian fetal clock begins to exhibit intrinsic rhythmicity in terms of molecular clockwork only around birth and early postnatally, the phase of the new formation and appearance of rhythms in the fetal SCN is set by the maternal SCN early in the prenatal period. In mammals, the development of peripheral clocks depends on the maturation of the organ housing the clock as well as the maturation of the molecular clockwork. Thus, the first appearance of molecular oscillations is highly organ-specific [23].

### 2.4. Maternal Circadian Signals: Melatonin and Glucocorticoid

Among the maternal circadian signals likely to impact fetal rhythmicity, melatonin and GCs appear to play key roles. Maternal melatonin and GCs can transverse the placenta and so they can provide temporal signals to the fetus. Maternal melatonin signals play key roles in establishing and entraining fetal circadian clocks [24]. CLOCK/BMAL1 heterodimers interact physically and acetylate glucocorticoid receptors (GRs), thereby reducing their affinity to glucocorticoid responsive elements (GREs) and their translocation into the nucleus. CRY1 and CRY2 can interact with the C-terminal domain of GR in a ligand-dependent fashion, repressing the GR-mediated transactivation of certain target genes [26]. Rhythmic GC and melatonin secretion in human infants appear at three and nine weeks of age, respectively [24,27].

### 2.5. Melatonin in Pregnancy and the Fetus

Melatonin is primarily secreted by the pineal gland at night. Tryptophan is a precursor for melatonin biosynthesis. As well as the pineal gland, melatonin can be produced in many other organs, such as the retina, skin, and bone marrow [2]. Melatonin regulates variable physiological functions through the activation of two G protein-coupled receptors, melatonin receptor-1 (MT1) and -2 (MT2) [28]. Additionally, melatonin can interact with the nuclear hormone receptor family retinoid Z receptor (RZR)/retinoid acid receptor (ROR) for signal transduction [28]. Melatonin has pleiotropic biological functions that control circadian rhythms, redox homeostasis, inflammation, epigenetic regulation, and fetal development [4,29,30,31]. 

### 2.6. Placental Melatonin

During human pregnancy, the placenta secretes a plethora of hormones into the maternal circulation, which modulate the mother’s physiology and transfer the nutrients available to the fetus for growth [32]. Among these placental hormones, melatonin plays critical roles in driving maternal physiological adaptations and providing beneficial effects for both the mother and the fetus [33]. Placenta-derived melatonin acts as an autocrine, paracrine, and endocrine hormone in a non-circadian fashion [34]. In addition to the source of melatonin, the placental villous trophoblasts express melatonin receptors [33]. Placenta-derived melatonin not only acts with the MT1 and MT2 receptors but also directly scavenges free radicals, which reduce oxidative damage to placental tissues [33,34]. Maternal pineal melatonin can pass the placenta and transfer light signals to the fetus [35,36]. Thus, photoperiodic information perceived by the mother can be transferred to the fetus to synchronize fetal circadian rhythms via maternal melatonin rhythms [36]. Melatonin receptors are present in many areas of the human fetal brain [37]. Therefore, maternal and placental melatonin presumably play roles in the early stage of fetal development. During the neonatal period, the production of melatonin by the pineal gland is activated after birth, although it lacks the rhythmic secretion of melatonin until nine weeks of age in humans [28]. 

### 2.7. Glucocorticoids in Pregnancy and the Fetus

The SCN stimulates the release of GCs in a light-dependent manner, leading to a rhythmicity that peaks in the early morning just prior to lights-on and decreases throughout the day in humans [18]. The circadian GC rhythm is implicated in the coordination of clock function in central and peripheral tissues [38]. At the molecular level, the circadian clock and GCs relay on two parallel transcriptional–translational feedback loops that modulate each other [25]. The GC–GR complex binds glucocorticoid responsive elements (GREs) in the promoter region of several clock genes and various clock-controlled genes. Conversely, CLOCK/BMAL1 heterodimers that are active during the night interact physically and acetylate GR, thereby reducing its affinity to GREs and its translocation into the nucleus. Additionally, CRY1 and CRY2 can interact with the C-terminal domain of GR in a ligand-dependent fashion, repressing the GR-mediated transactivation of certain target genes. Furthermore, REV-ERBα, which is active during the day, acts as an inhibitor of BMAL1 expression, thus stabilizing the nuclear localization of GR, reinforcing its transcriptional activity. Through this complex network of interactions, GCs and the clock machinery can mutually regulate each other. Additionally, GCs have been reported to influence melatonin production through the regulation of the nuclear factor kappa-light-chain-enhancer of activated B cells (NFκB) transcriptional program [39].

Excess or deficient GC signaling during developmental windows may disrupt the developmental trajectory of the fetus, leading to permanent negative consequences [40]. Glucocorticoid receptors (GRs) are expressed in most fetal tissues and the placenta and are crucial for survival [41]. Although gestational GC levels show a strong circadian variation, it is not translated to the embryo. Fetal GC concentrations can be maintained at a stable level due to GC being inactivated by the placental GC barrier [42]. However, in stressful situations, an excessive level of GCs can saturate this barrier and reach fetal tissues, interfering with the developmental programming of the circadian clock and stress system [43]. As a result, increased DNA methylation in the GR promoter and reduced expression of GR have been shown in the hippocampus [44]. Such epigenetic modifications have been proposed as a possible underlying mechanism for altered regulation of the HPA axis. 

## 3. Maternal Chronodisruption and Offspring Health

### 3.1. Human Studies for Programming of Adult Diseases Related to Maternal Chronodisruption

Epidemiologic studies have revealed an association between gestational chronodisruption and adverse pregnancy outcomes [45,46,47,48,49,50,51,52,53]. The disruption of circadian rhythms in pregnant women can occur through shift work [45,46,47], jet travel across time zones [51,52], or exposure to light at night [48]. Several observational studies suggest that pregnant night workers might have an increased risk of miscarriage [48], hypertensive disorders of pregnancy [49], and preterm delivery [50]. Pregnant flight attendants were shown to have a higher risk of spontaneous abortion and miscarriage when compared to flight attendants who did not work during their pregnancy [51,52]. A meta-analysis of 62 studies with 196,989 women showed that working rotating shifts is associated with preterm delivery, an infant small for gestational age, preeclampsia, and gestational hypertension [53]. Additionally, an association between pregnant women working fixed night shifts and increased odds of preterm delivery and miscarriage was shown [53]. 

Nevertheless, little is known about whether maternal circadian rhythm disruption impairs offspring health in adulthood in human studies. A recent study showed that male offspring aged 9 to 16 years born to shift working mothers had higher awakening cortisol (natural GC) levels, followed by a steeper early decline and a flatter late decline (between 4 and 16 h after awakening) compared with those born to mothers who did not work night shifts [47]. These findings suggest that maternal rotating night shift work influences the circadian rhythm of GC secretion in young adult offspring. A case series study reported that newborns born to night workers had lower Apgar scores and breastfeeding difficulty, indicating worse outcomes [54]. Another report demonstrated that there is no association between nightshift work before or during pregnancy and mental health disorders in young adult offspring [55]. Given that only a few human studies are available on this issue and that these observational studies cannot directly establish a causal relationship between maternal chronodisruption and lifelong health in the offspring, animal models are of great importance to identify which mechanisms underlying maternal circadian disruption may influence the programming of offspring phenotypes and lead to the development of specific preventive interventions.

### 3.2. Animal Models of Maternal Chronodisruption

Table 1 summarizes various animal models utilized to investigate the relationship between maternal circadian rhythm disruption and offspring health [56,57,58,59,60,61,62,63,64,65,66,67,68,69,70,71,72,73,74]. Of note is that not only constant light, but also diurnal light deficiency and/or continuous darkness, are disruptive for the circadian system. Several conditions related to diurnal light deficiency, such as living at high latitudes [75] and vitamin D deficiency [76], have been linked to chronodisruption. Additionally, serotonin, the precursor of melatonin, was reported to adjust circadian rhythms through serotonergic afferents [77]. In the current review, we mainly focused on the commonly used animal models for studying maternal chronodisruption, and, for the sake of brevity, we have restricted the presented data to constant light, chronic photoperiod shift, pinealectomy, and glucocorticoid exposure.

**Figure 2:**
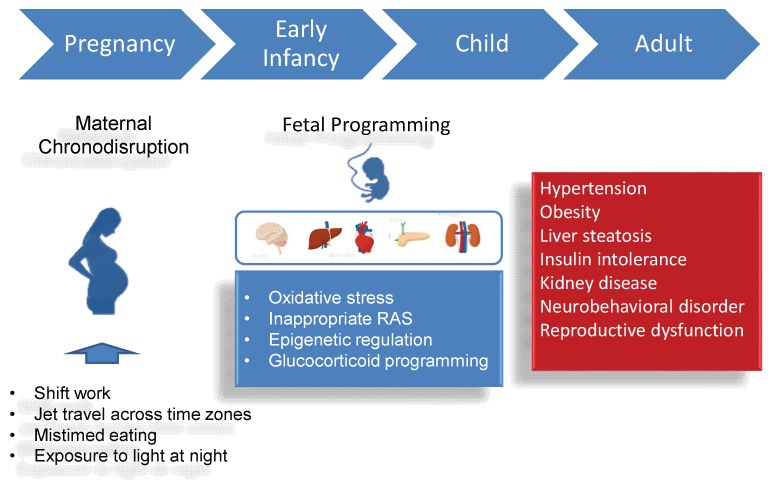
Schematic illustration of the association between maternal chronodisruction, fetal programming, and increased vulnerability to many adult disease.

The most commonly used animal species include rodents, sheep, and pigs. Light is known to promote sleep in nocturnal species and alertness in day-active animals. Thus, nocturnal (e.g., rodents) and diurnal (e.g., sheep and pigs) animals might differ in their temporal activity patterns and their circadian systems in response to constant light (or constant dark) exposure. As light is the principal environmental signal for the circadian system, exposing an animal to continuous constant light can disrupt the SCN-based clock and produce arrhythmic patterns of running-wheel activity. Adult offspring exposed to constant light prenatally have been reported to have an increased risk for developing hypertension [56], altered behaviors [57], and impaired cognition function [58]. These adverse outcomes are related to dysregulated melatonin signaling [57] and hippocampal clock gene expression [58]. Another approach is chronic photoperiod shift (CPH). Chronic phase shifts of the photoperiod throughout pregnancy programs adult offspring to display impaired endocrine, cardiovascular, and metabolic function [59,60]. Additionally, CPH during pregnancy has been reported to induce behavior changes characterized as hyperactivity and social avoidance in young adult rats [61]. Furthermore, gestational CPH has been reported to disrupt the peripheral liver clock [62]. Maternal pinealectomy results in melatonin deficiency in pregnant mothers. This model can cause negative outcomes in reproductive [63], neuropsychiatric [64], and metabolic function in adult offspring [65]. Finally, in utero exposure to the synthetic GC analogs dexamethasone (DEX) or betamethasone has been shown to induce chronodisruption as well as a wide range of adult diseases. Prenatal DEX exposure was reported to induce arrhythmic glucocorticoid secretion and an absence of circadian oscillations in hippocampal clock gene expression in adult offspring [66]. Moreover, antenatal GC exposure is related to hypertension [67,73], liver steatosis [69,72], hippocampal lesions [70], kidney disease [71,73], obesity [72], and an impaired HPA axis [74]. Although SCN ablation [78], timed food access [79], and genetic manipulation (e.g., CLOCK mutant mice) [80] have been employed to disrupt circadian rhythms in pregnant animals, their long-term effects on offspring have not been studied yet. To sum up, programming effects of maternal chronodisruption have been reported in rodents ranging from 1 week to 12 months of age, which is roughly equivalent to human ages from infancy to middle adulthood. However, future studies into the long-term offspring outcomes of maternal circadian disruption are still urgently warranted. The associations between maternal chronodisruption and the risks for many adult diseases are illustrated in Figure 2.

### 3.3. Mechanisms Underlying Maternal Chronodisruption-Induced Programmed Diseases

So far, the common mechanisms involved in the developmental programming of adult chronic diseases remain undetermined. However, emerging evidence has provided crucial insight into the pathways involved, including oxidative stress [81,82], inappropriate activation of the renin–angiotensin system (RAS) [83], epigenetic regulation [84], and glucocorticoid programming [40,85] (Figure 2). Notably, extensive experimental animal studies have demonstrated interplay between circadian rhythm disruption and the above-mentioned mechanisms [86,87,88,89]. 

First, the fetus is not sufficient to overcome reactive oxygen species (ROS) overproduction, due to its low-antioxidant capacity. In response to adverse in utero environments, the development of the fetus is thus vulnerable to oxidative stress damage [82,90]. Melatonin is not only a hallmark of circadian rhythm functionality but is also a natural antioxidant [91]. Thus, there is a close inter-relationship between oxidative stress and chronodisruption via melatonin signaling in a variety of diseases. As we previously reviewed [81], various early-life insults enable the induction of developmental programming linked to oxidative stress, such as maternal undernutrition [92], maternal diabetes [93,94], maternal exposure to ethanol [95], maternal inflammation [96], glucocorticoid exposure [68,97], preeclampsia [98], prenatal hypoxia [99], and a maternal high methyl-donor diet [100]. Conversely, maternal melatonin therapy showed a protective role that reverses the programming processes in models of maternal caloric restriction [92], N^G^-nitro-L-arginine-methyl ester (L-NAME) induced preeclampsia [98], a maternal high-fructose diet [101], a maternal high methyl-donor diet [100], and prenatal DEX exposure [70]. Considering the interplay between melatonin and oxidative stress, targeting circadian melatonin signals to counteract oxidative stress and protect against adult diseases of developmental origin, deserves further elucidation. 

Secondly, RAS is a well-known hormonal cascade that controls kidney development and blood pressure [102]. In humans, circulating renin, angiotensin (Ang) II, and aldosterone rhythms have an acrophase in early morning [87]. In nocturnal rat species, the activity of the RAS also has a circadian rhythm, with a diurnal acrophase [103]. In melatonin-deficient hypertension [104], the classical angiotensin-converting enzyme (ACE)–Ang II–angiotensin type 1 receptor (AT1R) axis is activated. In the constant light exposure model, the development of hypertension in adult rat offspring is related to activation of the RAS [56]. Various adverse conditions in utero have been reported to activate the classical RAS axis, resulting in renal programming and consequent hypertension in later life [105,106]. Apart from the classical RAS axis, the ACE2–angiotensin (1–7)–Mas receptor axis is also involved in developmental programming [107], by opposing many actions of Ang II on AT1R. Gestational melatonin use has been reported to block the activation of the classical RAS cascade and prevent the development of hypertension in adult offspring in several animal models, including maternal constant light exposure [56,108] and the prenatal GC exposure model [68]. On the other hand, maternal melatonin therapy can activate the ACE2–angiotensin (1–7)–MAS axis by the induction of renal *Agtr1b* and *Mas1* expression to prevent hypertension in adult male offspring programmed by prenatal GC exposure plus post-weaning from a high-fat diet [109]. Therefore, the results from these studies suggest that RAS may be an underlying mechanism involved in hypertension programmed by maternal circadian rhythm disruption.

Third are studies of crosstalk between circadian rhythms and epigenetic regulation [88]. Epigenetic mechanisms such as post-translational modification of histones, DNA methylation, and RNA interference play key roles in gene regulation [30]. Histone modifications, including acetylation–deacetylation and methylation–demethylation, are involved in regulating the expression of transcription factors of Clock genes [88,110]. Interestingly, CLOCK possesses histone acetyltransferase activity [111]. On the other hand, many epigenetic modification enzymes are rhythmically expressed [88]. Thus, these observations support the view that the circadian clock can directly regulate epigenetic modification enzymes and that these enzymes, in turn, contribute feedback to the circadian clock, contributing to the mutual regulation of oscillators. On the other hand, the circadian signal melatonin is also involved in epigenetic regulation. In a prenatal GC model [70], maternal melatonin therapy restored reelin mRNA expression levels by reducing DNA methyltransferase 1 (DNMT1) expression. Additionally, melatonin and trichostatin A (a histone deacetylase [HDAC] inhibitor) have similar beneficial effects on hypertension programmed by GC exposure [112], suggesting that melatonin acts as a HDAC inhibitor. These observations support the view that epigenetic regulation may contribute to the development of hypertension programmed by maternal circadian rhythm disruption. 

Last, emerging evidence supports the interplay between glucocorticoid programming and chronodisruption on developmental programming [85,89]. A developing fetus is prone to exposure to excessive glucocorticoids through exogenous administration (e.g., preterm birth) or through excess maternal corticosteroids (e.g., stressed pregnancies). The HPA axis is particularly susceptible to developmental programming by GCs, impairing a variety of organ systems, such as the kidneys [68,71,72], liver [69,72], central nervous system [70], and endocrine system [72,74]. As rhythmic GCs coordinate central and peripheral clocks, prenatal GC exposure-induced obesity and hypertension are relevant to altered clock genes in peripheral tissues [67]. Conversely, melatonin use in pregnancy has been reported to protect prenatal GC-induced programming of hypertension [113] and cognition deficit [70]. 

## 4. Targeting on Light and Circadian Signaling Pathway as a Reprogramming Therapy

### 4.1. Light and Circadian Signaling-Related Therapy in Human Diseases 

Light therapy has been evaluated in healthy individuals undergoing shift work, jet travel, and space flights [8,9]. Although lighting based on light-emitting diodes has the potential to improve human health [114], its clinical application for specific human diseases still has a long way to go. Photobiomodulation (PBM) uses low levels of visible or near-infrared light to heal and stimulate tissue [115]. So far, PBM has been applied to treat several human diseases such as stroke, Parkinson’s disease, Alzheimer’s disease, traumatic brain injury, and psychiatric disorders [115]. Although there have been some studies relevant to light therapy [116,117] in pregnancy, none of them have focused on offspring outcomes in adulthood. In the area of complementary and alternative medicine, several suggested therapies for insomnia might be applied for treating chronodisruption, despite not yet being recommended as standard therapies [118]. These therapies include herbal medicine, acupuncture, acupressure, aromatherapy, foot reflexology, music therapy, and yoga. Four herbal medicines—valerian, chamomile, kava, and wuling—have been examined regarding their clinical efficiency for insomnia in a meta-analysis study, while there is insufficient evidence to support the use of herbal medicine for insomnia [119]. So far, whether complementary and alternative medicines can be applied in maternal chronodisruption to improve offspring outcomes remains largely unknown.

Furthermore, circadian melatonin signaling has been used to treat a variety of human diseases [11,120,121,122]. Oral melatonin dosages ranging from 0.3 to 1600 mg daily have been reported to be relatively safe for humans. [123]. Although emerging evidence from animal models of developmental programming suggests that melatonin use in pregnancy and lactation has reprogramming effects that prevent DOHaD-related disorders [31,124], no clinical trials of melatonin in pregnant women have been conducted to assess its use and safety. Another circadian signal, GC, has been well used in human diseases. Antenatal GC intake is recommended to accelerate fetal lung maturation. Nevertheless, prenatal GC intake induces many long-term negative outcomes that greatly influence its therapeutic potential in DOHaD-related disorders. 

### 4.2. Melatonin as a Reprogramming Therapy in Animal Models 

While current medical treatment focuses on high-risk individuals in adulthood, DOHaD concepts offer a “reprogramming”’ strategy to prevent the development of adult diseases during early life [125]. So far, melatonin seems to be the only reprogramming therapy that is likely to catch the attention of researchers. The overview of experimental studies in Table 2 illustrates data documenting the reprogramming effects of melatonin treatment in the pregnancy and/or lactation period in animal studies. We only considered studies reporting offspring outcomes starting from childhood in the present review.

As shown in Table 2, rodents have been the dominant animal species used. Various developmental programming models have been examined, including maternal caloric restriction [92], L-NAME induced preeclampsia [98], a maternal high-fructose diet [101], maternal hyperhomocysteinemia [126], maternal phenytoin exposure [127], a maternal high-fructose diet plus a post-weaning high-salt diet [128], maternal constant light exposure [56,108], a maternal high methyl-donor diet [100], prenatal GC exposure [69,112,129,130], and maternal hypermethioninemia [131]. These environmental insults lead to adverse offspring outcomes including cognition deficits [126], neurobehavioral dysfunction [106,127], hypertension [56,92,98,100,101,113], and liver steatosis [129,130]. All of these adverse offspring phenotypes can be prevented, or at least moderated, by maternal melatonin treatment. It is notable that melatonin has pleiotropic biological functions in pregnancy; these beneficial reprogramming effects might not be directly attributed to its regulation of the circadian rhythm [35]. Therefore, additional studies are warranted to elucidate the underlying mechanisms of melatonin and determine appropriate therapeutic windows and ideal doses of melatonin before clinical translation.

## 5. Conclusions

This review highlights the importance of considering environmental light and maternal circadian rhythms during pregnancy. Given the increasing incidence of shift work, jet travel across time zones, and mistimed eating in our modern society, large numbers of pregnant women are exposed to adverse environmental conditions. These suboptimal maternal conditions have implications for the developing fetus. Maternal chronodisruption affects not only central and peripheral circadian clocks but also a range of endogenous circadian signals including melatonin and GC secretion. Despite most programming effects and reprogramming approaches being examined in animal models, these observations have important translational applications, as they open a new avenue for testing the prevention of adult disease by targeting light and circadian signaling pathways in pregnant women with disrupted circadian rhythms. 

## Figures and Tables

**Table 1:** Commonly used animal models for studying the impacts of maternal chronodisruption on offspring health.

Model	Technique	Impacts on Offspring Health
Constant light	24-h constant light exposure during pregnancy	Induced hypertension in 12-week-old rat offspring [56] Induced behavior changes and melatonin signaling dysregulation in 90-day-old rat offspring [57] Impaired cognition function and altered hippocampal clock gene expression in 90-day-old rat offspring [58]
Chronic photoperiod shift	Repeated photoperiod shifts during pregnancy	Induced hyperinsulinemia and insulin intolerance in 12-month-old female rat offspring [59] Altered endocrine, cardiovascular, and metabolic function in 90-day-old rat offspring [60] Induced behavior changes with hyperactivity and social avoidance in 60-day-old rat offspring [61] Disrupted daily rhythms in hepatic clock genes in 3-month-old rat offspring [62]
Pinealectomy	Surgical removal of pineal gland	Altered seasonal variations of reproductive hormones in 60-day-old rat offspring [63] Increased depressive-like responses in adult swine offspring [64] Induced glucose intolerance in 18-week-old rat offspring [65]
Glucocorticoid exposure	Prenatal dexamethasone treatment	Induced depression-like behavior, arrhythmic glucocorticoid secretion, and absent circadian oscillations in hippocampal clock gene expression in 12-month-old mice offspring [66] Altered clock genes in adipose tissue and enhanced obesity, insulin dysregulation, and hypertension in 6-month-old rat offspring [67] Induced hypertension in 16-week-old rat offspring [68] Induced liver steatosis in 7-day-old offspring [69] Altered hippocampal morphology in 16-week-old rat offspring [70] Altered transcriptome in 16-week-old offspring kidney [71]
	Prenatal betamethasone treatment	Induced obesity and liver steatosis in 10-year-old baboons [72] Induced hypertension and renal dysfunction in 1.5-year-old sheep [73] Altered hippocampal expression of HPA-related genes in 3.5-year-old sheep [74]

Studies tabulated according to animal models and techniques. HPA: hypothalamic–pituitary–adrenal.

**Table 2:** Reprogramming effects prevented by melatonin.

Animal Models	Route of Administration	Reprogramming Effects
Maternal caloric restriction	Drinking water	Prevented hypertension in 12-week-old rat offspring [92]
Maternal L-NAME exposure	Drinking water	Prevented hypertension in 12-week-old rat offspring [98]
Maternal high-fructose diet	Drinking water	Prevented hypertension in 12-week-old rat offspring [101]
Maternal hyperhomocysteinemia	Subcutaneous injection	Prevented cognition deficit in 75-day-old rat offspring [126]
Maternal phenytoin exposure	Drinking water	Protected neurobehavioral dysfunctions in 12-week-old rat offspring [127]
Maternal constant light exposure	Drinking water	Prevented hypertension in 12-week-old rat offspring [56]
	Drinking water	Protected anxiety-like and sexual behaviors in 16-week-old rat offspring [106]
Maternal high methyl-donor diet	Drinking water	Attenuated hypertension and altered renal transcriptome in 12-week-old rat offspring [100]
Maternal high-fructose diet plus post-weaning high-salt diet	Drinking water	Attenuated hypertension in 12-week-old rat offspring [128]
Prenatal GC exposure	Drinking water	Protected hippocampal morphology in 16-week-old rat offspring [70]
	Drinking water	Prevented hypertension and increased nephron number in 16-week-old rat offspring [113]
	Drinking water	Protected liver steatosis in 16-week-old rat offspring [129]
Prenatal GC exposure plus post-weaning high-fat diet	Drinking waterDrinking water	Prevented hypertension in 16-week-old rat offspring [109] Protected liver steatosis in 6-month-old rat offspring [130]
Maternal hypermethioninemia	Subcutaneous injection	Protected impaired recognition and neurons in 30-day-old rat offspring [131]

L-NAM E = N^G^-nitro-L-arginine methyl ester. GC = glucocorticoid.

## Abbreviations

ACE	Angiotensin-converting enzyme
AT1R	Angiotensin type 1 receptor
BMAL1	Brain and muscle aryl-hydrocarbon receptor nuclear translocator-like 1
CLOCK	Circadian locomotor output cycles kaput
CPH	Chronic photoperiod shift
DEX	Dexamethasone
DNMT	DNA methyltransferases
DOHaD	Developmental origins of health and disease
GC	Glucocorticoid
HDAC	Histone deacetylase
HPA	Hypothalamic–pituitary–adrenal
L-NAME	N^G^-nitro-L-arginine-methyl ester
MT	Melatonin receptor
PBM	Photobiomodulation
RAS	Renin-angiotensin system
RHT	Retinohypothalamic tract
ROS	Reactive oxygen species
SCN	Suprachiasmatic nucleus
